# Identifying Live Bird Markets with the Potential to Act as Reservoirs of Avian Influenza A (H5N1) Virus: A Survey in Northern Viet Nam and Cambodia

**DOI:** 10.1371/journal.pone.0037986

**Published:** 2012-06-04

**Authors:** Guillaume Fournié, Javier Guitian, Stéphanie Desvaux, Punam Mangtani, Sowath Ly, Vu Chi Cong, Sorn San, Do Huu Dung, Davun Holl, Dirk U. Pfeiffer, Sirenda Vong, Azra C. Ghani

**Affiliations:** 1 Veterinary Clinical Sciences Department, Royal Veterinary College, University of London, United Kingdom; 2 UR Animal et gestion intégrée des risques, Centre de Cooperation Internationale en Recherche Agronomique pour le Développement, Montpellier, France; 3 Infectious Disease Epidemiology Unit, London School of Hygiene and Tropical Medicine, London, United Kingdom; 4 Epidemiology Unit, Institut Pasteur, Phnom Penh, Cambodia; 5 National Institute of Animal Science, Ha Noi, Viet Nam; 6 National Veterinary Research Institute, Ministry of Agriculture, Fisheries and Forestry, Phnom Penh, Cambodia; 7 Department of Animal Health, Ha Noi, Viet Nam; 8 MRC Centre for Outbreak Analysis and Modelling, Department of Infectious Disease Epidemiology, Imperial College, London, United Kingdom; Harvard School of Public Health, United States of America

## Abstract

Wet markets are common in many parts of the world and may promote the emergence, spread and maintenance of livestock pathogens, including zoonoses. A survey was conducted in order to assess the potential of Vietnamese and Cambodian live bird markets (LBMs) to sustain circulation of highly pathogenic avian influenza virus subtype H5N1 (HPAIV H5N1). Thirty Vietnamese and 8 Cambodian LBMs were visited, and structured interviews were conducted with the market managers and 561 Vietnamese and 84 Cambodian traders. Multivariate and cluster analysis were used to construct a typology of traders based on their poultry management practices. As a result of those practices and large poultry surplus (unsold poultry reoffered for sale the following day), some poultry traders were shown to promote conditions favorable for perpetuating HPAIV H5N1 in LBMs. More than 80% of these traders operated in LBMs located in the most densely populated areas, Ha Noi and Phnom Penh. The profiles of sellers operating at a given LBM could be reliably predicted using basic information about the location and type of market. Consequently, LBMs with the largest combination of risk factors for becoming virus reservoirs could be easily identified, potentially allowing control strategies to be appropriately targeted. These findings are of particular relevance to resource-scarce settings with extensively developed LBM systems, commonly found in South-East Asia.

## Introduction

First detected in 1996 [1], highly pathogenic avian influenza virus subtype H5N1 (HPAIV H5N1) has spread across 3 continents and is now considered to be endemic in several South-East Asian countries and Egypt. Due to its potential to recombine with human influenza strains to produce highly virulent reassortants [2], ongoing circulation of HPAIV H5N1 continues to be a major public health concern.

Live bird markets (LBMs), in which the virus has been frequently detected in both disease-endemic and epidemic regions [3], [4], [5], are suspected to play a major role in the epidemiology of HPAIV H5N1 [6], [7]. The LBM system provides consumers with freshly slaughtered birds. It is a dead-end for poultry, but not necessarily for viruses. LBMs have been shown to contribute to the spread of, and the possible maintenance of, HPAIV H5N1 within the poultry sector [8]. Zoonotic transfer to humans has also been documented in LBMs [9], [10].

Birds are introduced into LBMs daily and stocked at a high density. If these birds remain in the market for a sufficient time to become infected and transmit virus to susceptible birds, LBMs would offer optimal conditions for amplifying and sustaining virus circulation and could, thus, become viral reservoirs themselves [11].

Given the range of species present in typical LBMs and the variety of detected influenza viruses [12], [13], [14], [15], [16], [17], [18], [19], LBMs could potentially act as drivers of viral evolution, promoting the emergence of new variants. Identifying those LBMs that could act as viral reservoirs is, therefore, crucial for improving surveillance and control.

Although the potential for an LBM to become a viral reservoir is determined by the management practices of poultry traders [11], an understanding of these practices in high risk areas is lacking. In response to this need, a cross-sectional survey was conducted in northern Viet Nam and Cambodia to assess whether traders of live poultry engaged in practices that could sustain virus circulation in LBMs. Viet Nam is one of the most severely affected countries in the current HPAI H5N1 pandemic [20], with the disease considered to be endemic in both northern and southern Viet Nam. Only sporadic outbreaks have been reported in Cambodia [20]. However, the reporting of isolated human cases without prior notification of poultry outbreaks [21] suggests that widespread, undetected virus circulation and persistence of HPAIV H5N1 in the Cambodian poultry population cannot be ruled out. In both countries, LBMs are common and potentially involved in virus spread.

## Methods

### Sample Selection

An LBM for this study was defined as an open space with 2 or more traders selling live poultry at least once per week and with official government authorization to do so. “Live poultry” referred to finished birds, meaning birds intended to be slaughtered and eaten by the end-user.

Although LBMs are numerous in Viet Nam and Cambodia, the live poultry trade is only a small, irregular activity in most markets, necessitating a purposive sampling strategy. In the selected areas, only the largest LBMs in terms of the number of poultry sold were eligible. The selection of Cambodian LBMs was based on a previous cross-sectional survey on commercial poultry movements conducted in 2006–2007 [22]. Eight Cambodian LBMs with the highest volumes of poultry sales were recruited.

Data on the frequency and volume of live poultry sales in northern Vietnamese LBMs were not available. Study provinces were selected based on demographic features rather than outbreak reports, given that most disease events were presumed to be undetected [23], [24], [25]: Ha Noi, the most densely populated province in northern Viet Nam [26], and Bac Giang, a rural province with a large poultry population [27]. A snow-ball sampling approach was adopted.

A first set of major LBMs were identified in each study area through meetings with trade and veterinary service officers. In these LBMs, traders were asked to identify the other LBMs of which they were aware and to rank them according to the number of live poultry sellers. These identified LBMs were then integrated into the survey and their traders were in turn asked to name the LBMs that they considered to be the biggest.

As there are 4 times as many people in Ha Noi as in Bac Giang [26], the number of LBMs was also expected to be much higher, with traders likely to be aware only of markets located in the immediate vicinity of those from which they trade. For this reason, the snowball approach described above was applied only at the province level in Bac Giang, but also at the lower administrative level, the district level, in Ha Noi.

**Figure 1:**
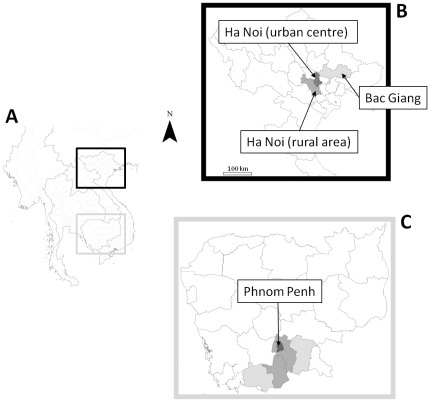
Study areas in northern Viet Nam and Cambodia. (A) Map of South-East Asia. (B) Map of northern Viet Nam. Dark grey: former Ha Noi province (number of LBMs n = 14); medium grey: former Ha Tay province (n = 7); light grey: Bac Giang province (n = 9). (C) Map of Cambodia. Dark grey: Phnom Penh province (n = 3); medium grey, from left to right: Takeo province (n = 1) and Kandal province (n = 2, on the right); light grey, from left to right: Kampot province (n = 1) and Prey Veng province (n = 1). Province boundaries are shown.

The present day province of Ha Noi is the result of the recent merging of an urban centre (former Ha Noi province) and a rural area (former Ha Tay province and a district from Vinh Phuc province). After exclusion of districts where live poultry marketing was prohibited, 4 of 5 districts in the urban centre and 2 of 13 districts in the rural area were randomly selected. Meetings held with Ha Noi veterinary services also identified the 4 main LBMs supplying the province, which were then integrated into the survey. In total, 30 markets were recruited in northern Viet Nam. Assuming that each commune (administrative division of district) had 1 or 2 LBMs, the LBM sampling rate per district ranged between 3% and 25%. Study areas are shown in Fig. 1.

To participate in the survey, traders in the markets had to sell or purchase birds in an LBM at least 1 day per month. Live poultry traders consisted of sellers and middlemen. A seller was defined as selling live poultry mostly to the end-user (e.g. consumers, restaurants) or to another trader who would then sell the poultry at another location. A middleman was defined as selling live poultry to mostly sellers, or purchasing live poultry from sellers and then re-selling them at another location (e.g. market, restaurants). All traders that were present during the visit of the selected LBMs were interviewed, except in the largest LBM in Viet Nam where half were interviewed. Of note, in all other LBMs, the numbers of interviewed sellers corresponded well to numbers indicated by market managers.

The study periods, April–May 2009 in Viet Nam and June–July 2009 in Cambodia, did not include any seasonal festivals which could potentially influence live poultry sale patterns.

### Procedures

Three standardized questionnaires were designed for interviewing market managers, sellers, and middlemen. The questionnaires were translated into Vietnamese and Khmer and administered by trained interviewers. All questionnaires were piloted in Vietnamese LBMs that were not included in the survey. Informed oral consent was sought prior to interviewing. At the conclusion of each interview, the completed questionnaire was reviewed by the author for missing, unclear, or inconsistent answers. Questions for which the accuracy of the answer was doubtful were posed a second time. Market observations, such as counting the number of birds offered for sale and the number of birds left unsold at the end of the market day, were made during the visits. The interviewees' answers were consistent with these observations.

### Variables

LBMs were described based on demographic features provided by market managers: days of the month and time of the day during which LBMs were open, average number of sellers, and seasonal variations. Sellers were classified as retailers or wholesalers if they reported selling most birds to consumers or traders, respectively. Markets were classified as either retail or wholesale markets if more than two thirds of sellers were retailers or wholesalers. Markets that did not fall into one of these categories were classified as mixed.

Traders' practices likely to influence the sustainability of virus circulation in LBMs were recorded; essentially, those that could impact the length of time during which birds remained in the market chain and the contact rate between birds. These factors included the number of days during which traders were active, and the length of time they spent at market in a day. The number of poultry sold within a day, and the type of poultry were also considered. Indeed, the susceptibility of chickens and ducks (muscovy or mallard duck derived breeds) to infection is known to differ [28]. Moreover, the supply management, and frequency and quantity of the surplus (unsold poultry reoffered for sale the following day) could impact on the length of time that poultry spent at market. The surplus frequency referred to the proportion of days traders reported having had surplus and the surplus volume to the proportion of birds left unsold when having a surplus. The surplus frequency was captured in 2 ways: the usual surplus frequency, recorded as a categorical variable (categories: never, sometimes, half of the time, often, always), and the surplus frequency in the last week, defined as the proportion of days with a surplus out of the number of trading days during the week preceding the interview. Since both variables were highly correlated (correlation ratio = 0.84), only the surplus frequency in the past week was kept in the analysis. The management of the supply described the frequency at which poultry were purchased, and whether poultry were purchased the day before being offered for sale and kept overnight at traders' homes. Changes in trade practices during festivals were also described.

Information regarding the origin of poultry and the number and type of visited farms and LBMs was also collected, as these contacts could influence the likelihood of spreading infection into and out of LBMs.

### Statistical Analysis

Questionnaire data were entered in Microsoft Access 2007® (Microsoft Corp., Redmond, WA, USA) database. The accuracy of the data entry was verified by cross-checking each questionnaire with the recorded entry.

Numerical variables were summarized as medians with inter-quartile ranges (IQR); binary and categorical variables as frequencies and percentages.

Multivariate analysis was performed to describe trader profiles, which were based on poultry management practices that could increase the risk of sustained virus circulation in LBMs (Table 1). As variables were both numerical and categorical, factor analysis for mixed data (FAMD) [29], [30] was used. This method allows a reduction in the dimensions of multivariate data, creating a smaller number of synthetic (and uncorrelated) factors accounting for most data variability. Further details are provided in Text S1.

**Table 1:** Poultry management variables included in the multivariate analysis.

Presence at market	
No. days traders sold poultry during one month	Numerical (in days)
Length of time spent at markets per day	Numerical (in hours)

Number and type of poultry sold	
Frequency of chicken sales	Ordered categorical (categories: never, sometimes, seasonal, often, always)
Number of chickens sold per day	Numerical
Frequency of duck sales	Ordered categorical (never, sometimes, seasonal, often, always)
Number of ducks sold per day	Numerical

Supply	
Supply management*	Categorical (No supply, Every time & Storage overnight, Every time & No storage overnight, Not every time & Storage overnight, Not every time & No storage overnight)

Surplus	
Surplus frequency (proportion of days with a surplus out of number of trading days during the previous week)	Numerical
Proportion of chickens that are unsold on a day with a surplus	Numerical
Proportion of ducks that are unsold on a day with a surplus	Numerical

* : For the “supply management” variable, “No supply” meant that traders sold their own stock only, “Every time” implied that traders were supplied with birds every time they went to market, and “Not every time” that they were supplied with birds but not every time they went to market. “Storage overnight” refers to traders keeping birds at home for at least one night before bringing them to markets; “No storage overnight” indicates that birds were either bought at markets or brought directly to markets.

Hierarchical cluster analysis (HCA) [31] was then used to group traders into clusters according to their level of similarity in the factors created by the FAMD. The Manhattan distance was used to assess the level of dissimilarity between 2 traders. The algorithm was agglomerative, and the Ward's criteria for linkage was adopted. Finally, a consolidation using the *k*-means algorithm was performed. FAMD and HCA were implemented in R 2.12.0 [32], using the package FactoMineR 1.15 [33].

## Results

There were 340 sellers and 221 middlemen interviewed in 30 Vietnamese LBMs, and 54 sellers and 30 middlemen in 8 Cambodian LBMs. The refusal rate was 8% among Vietnamese traders, with the principal reason being that they were too busy to participate. In Cambodia, only 2 (2%) people declined interviews.

LBM features are described in Table 2. LBMs in Bac Giang province in Viet Nam were either open every day (n = 4) or periodically (n = 5). Periodic markets were open 6 or 12 days per month. LBMs in Ha Noi province were grouped as either retail and mixed markets (n = 17), hosting from 2 to 13 sellers, or wholesale markets (n = 4), which were the largest markets, hosting from 30 to 80 sellers. Cambodian LBMs were grouped as either urban markets (n = 4), located in Phnom Penh or its close proximity, or peri-urban markets (n = 4), in other provinces. Urban markets were open throughout the day for 12–15 hours, whereas peri-urban markets were only open in the morning for 6 hours or less.

**Table 2:** Features of market groups.

	Vietnamese markets (n = 30)				Cambodian markets (n = 8)	
	Bac Giang periodic markets (n = 5)	Bac Giang everyday markets (n = 4)	Ha Noi retail and mixed markets (n = 17)	Ha Noi wholesale markets (n = 4)	Urban markets (n = 4)	Peri-urban markets (n = 4)
Average number of sellers	13 (8–14)	11 (6–25)	6 (2–13)	44 (30–80)	7 (4–19)	7 (4–8)
Length of time markets are open (in hours)	5 (4–6)	4 (3–7)	7 (4–14)	11 (5–24)	13 (12–15)	6 (4–6)
Market type						
Retail	2 (40%)	0 (0%)	15 (88%)	0 (0%)	3 (75%)	2 (50%)
Mixed	3 (60%)	2 (50%)	2 (12%)	0 (0%)	0 (0%)	0 (0%)
Wholesale	0 (0%)	2 (50%)	0 (0%)	4 (100%)	1 (25%)	2 (50%)
Periodicity						
Periodic	5 (100%)	0 (0%)	0 (0%)	0 (0%)	0 (0%)	0 (0%)
Everyday	0 (0%)	4 (100%)	17 (100%)	4 (100%)	4 (100%)	4 (100%)
Area						
Rural	1 (20%)	0 (0%)	2 (12%)	1 (25%)	0 (0%)	0 (0%)
Peri-urban	4 (80%)	4 (100%)	8 (47%)	2 (50%)	0 (0%)	4 (100%)
Urban	0 (0%)	0 (0%)	7 (41%)	1 (25%)	4 (100%)	0 (0%)

Data are median (Minimum-Maximum), or no. (%).

Most Vietnamese (80%, n = 340) and Cambodian (70%, n = 54) sellers reported having a surplus, at least occasionally. In contrast, most middlemen reported never having a surplus (Viet Nam: 76%, n = 221; Cambodia: 97%, n = 30). As they generally spent less than 1 hour in a LBM, middlemen kept their poultry in LBMs for only a very short time and were therefore excluded from the multivariate analysis.

Following the FAMD and the HCA, Vietnamese sellers were divided into 4 clusters and Cambodian sellers into 2 clusters. Tables 3 and 4 present the distribution of poultry management and contact features for each seller profile in Viet Nam and Cambodia, respectively. A description of the factors is provided in Text S1.

**Table 3:** Features of Vietnamese seller clusters.

	V.1 Farmers and irregular sellers (n = 43)	V.2 Medium-scale sellers with no or low surplus (n = 162)	V.3 Medium-scale sellers with high surplus (n = 71)	V.4 Large-scale sellers (n = 64)
Poultry management features				
Presence at market				
No. days/month	12 (5–12)	30 (24–30)	30 (23–30)	30 (28–30)
No. hours/day	3 (2–4)	4 (3–5)	5 (4–10)	11 (7–13)
Type of poultry sold				
Sellers trading only chickens	20 (47%)	51 (31%)	16 (23%)	30 (47%)
Sellers trading only ducks	3 (7%)	14 (9%)	9 (13%)	17 (27%)
Sellers trading chickens and ducks	20 (47%)	97 (60%)	46 (65%)	17 (27%)
Number of poultry sold				
No. chickens sold/day	7 (6–10)	20 (11–42)	10 (5–19)	200 (100–417)
No. ducks sold/day	6 (5–9)	15 (8–30)	9 (5–15)	142 (100–200)
Supply management				
No supply (farmers)	40 (93%)	4 (2%)	0 (0%)	0 (0%)
Every time & storage overnight	1 (2%)	49 (30%)	18 (25%)	4 (6%)
Every time & no storage overnight	2 (5%)	98 (60%)	21 (30%)	59 (92%)
Not every time & storage overnight	0 (0%)	11 (7%)	29 (41%)	1 (2%)
Not every time & no storage overnight	0 (0%)	0 (0%)	3 (4%)	0 (0%)
Surplus management				
Surplus frequency	0% (0%–25%)	14% (7%–40%)	100% (71%–100%)	29% (7%–57%)
Proportion of unsold chickens*	15% (8%–20%)	10% (7%–17%)	32% (22%–42%)	9% (6%–13%)
Proportion of unsold ducks*	9% (5%–13%)	6% (5%–12%)	17% (12%–25%)	7% (4%–10%)
Contact pattern features				
Origin of poultry				
Own flock	40 (93%)	4 (2%)	0 (0%)	0 (0%)
Only farmers	2 (5%)	93 (57%)	47 (66%)	45 (70%)
Only traders	0 (0%)	16 (10%)	16 (23%)	7 (11%)
Farmers and traders	1 (2%)	49 (30%)	8 (11%)	12 (19%)
Supplying farm size**				
Backyard (<50 birds)	2 (67%)	54 (38%)	17 (31%)	1 (2%)
Small commercial farms (50–500)	1 (33%)	74 (52%)	34 (62%)	33 (58%)
Large farms (>500)	0 (0%)	14 (10%)	4 (7%)	23 (40%)
No. suppliers***				
No. supplying farmers/day	-	3 (2–4)	2 (1–3)	2 (1–2)
No. supplying traders/day	-	3 (2–5)	3 (1–4)	1 (1–2)
Sellers visiting at least another market				
Yes	9 (21%)	78 (48%)	27 (38%)	2 (3%)
No	34 (79%)	84 (52%)	44 (62%)	62 (97%)

Data are median (inter-quartile range), or no. (%);

* : proportion of unsold chickens and ducks on a day with surplus;

** : the denominator is equal to the number of sellers supplied by farmers;

*** : only sellers supplied by each type of supplier are taken into account.

**Table 4:** Features of Cambodian seller clusters.

	C.1 Sellers with no or low surplus (n = 26)	C.2 Sellers with high surplus (n = 28)
Poultry management features		
Presence at market		
No. days/month	30 (30–30)	30 (30–30)
No. hours/day	4 (3–5)	11 (10–12)
Type of poultry sold		
Sellers trading only chickens	19 (73%)	13 (46%)
Sellers trading only ducks	0 (0%)	0 (0%)
Sellers trading chickens and ducks	7 (27%)	15 (54%)
Number of poultry sold		
No. chickens sold/day	25 (10–38)	35 (19–73)
No. ducks sold/day	8 (6–10)	5 (4–22)
Supply management		
No supply (farmers)	0 (0%)	0 (0%)
Every time & storage overnight	6 (23%)	1 (4%)
Every time & no storage overnight	20 (77%)	22 (79%)
Not every time & storage overnight	0 (0%)	1 (4%)
Not every time & no storage overnight	0 (0%)	4 (14%)
Surplus management		
Surplus frequency	0% (0%–13%)	100% (64%–100%)
Proportion of unsold chickens*	13% (11%–16%)	24% (20%–31%)
Proportion of unsold ducks*	0% (0%–0%)	19% (11%–24%)
Contact pattern features		
Origin of poultry		
Own flock	0 (0%)	0 (0%)
Only farmers	10 (38%)	4 (14%)
Only traders	10 (38%)	23 (82%)
Farmers and traders	6 (23%)	1 (4%)
Supplying farm size**		
Backyard (<50 birds)	16 (100%)	5 (100%)
Small commercial farms (50–500)	0 (0%)	0 (0%)
Large farms (>500)	0 (0%)	0 (0%)
No. suppliers***		
No. supplying farmers/day	5 (4–6)	6 (3–10)
No. supplying traders/day	3 (2–4)	2 (1–3)
Sellers visiting at least another market		
Yes	0 (0%)	0 (0%)
No	26 (100%)	28 (100%)

Data are median (inter-quartile range), or no. (%);

* : proportion of unsold chickens and ducks on a day with surplus;

** : the denominator is equal to the number of sellers supplied by farmers;

*** : only sellers supplied by each type of supplier are taken into account.

Vietnamese sellers in Cluster V.1 were farmers or occasional sellers. Most farmers' flocks consisted of 50–500 birds and were located in the market vicinity. They were characterized by an infrequent and short presence at market and a very low number of sales. Encompassing 48% (n = 340) of Vietnamese sellers, Cluster V.2 was composed of sellers trading larger volumes and more frequently than Cluster V.1 sellers. However, they spent little time in LBMs, with 60% (n = 162) only trading 4 hours or less per day. Their surpluses were also low, with the median frequency of having a surplus being 14% and the median proportion of unsold chickens and ducks being 10% and 6%, respectively. Cluster V.3 was also composed of regular sellers. Although their number of sales was slightly lower than Cluster V.2 sellers, they reported higher surpluses than traders in other clusters. The proportion of traders reporting a surplus every day was 51% (n = 71), and the median proportion of unsold chickens and ducks was 32% and 17%, respectively. Moreover, 45% (n = 71) of Cluster V.3 sellers were not supplied every day and 66% (n = 71) purchased birds the day before offering them for sale, keeping them overnight at home. These proportions were higher than in other clusters. For traders who were not supplied every day when operating at a market, part of the newly purchased birds were stored at home for 1 to several days before bringing them to market. Cluster V.4 included sellers spending more time at market, with 64% (n = 64) trading at least 10 hours per day, and selling substantially more poultry than other clusters. However, the median surplus frequency of 29% and the median proportion of unsold birds, less than 10% for chickens and ducks, were much lower than those reported by Cluster V.3 sellers.

Except for Cluster V.1, most Vietnamese sellers were supplied by farms, the majority of which were small commercial farms (50–500 birds). Cluster V.2 and V.3 sellers were also supplied by backyard farms (<50 birds), whilst Cluster V.4 sellers were also supplied by large farms (>500 birds). The number of sellers visiting several markets to purchase or sell poultry was higher in Cluster V.2 (48%, n = 162) than in Clusters V.3 (38%, n = 71) and V.4 (3%, n = 64).

**Figure 2:**
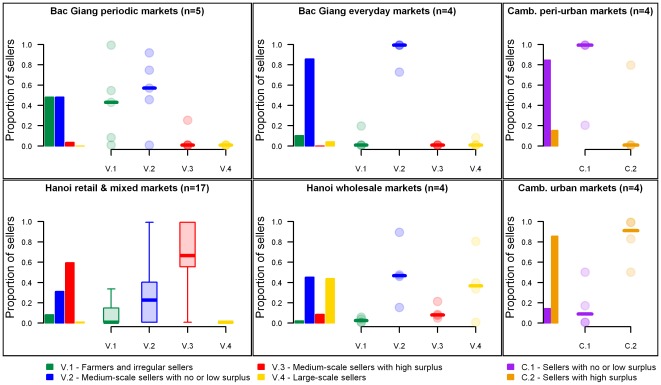
Distribution of seller clusters across markets and market groups. For each market group, a barplot (on the left) shows the proportion of its sellers in each cluster, and a plot (on the right) shows the distribution of its markets according to their proportion of sellers in each cluster. Where the number of markets in a group is greater than 5, box plots are shown; otherwise, each market (circle) and the median (line) are presented.

Whilst the proportion of Cluster V.2 sellers was high in all market groups, 74% (n = 43) Cluster V.1 sellers were found in Bac Giang markets, 80% (n = 71) Cluster V.3 sellers were in Ha Noi retail and mixed markets and 95% (n = 64) Cluster V.4 sellers were in Ha Noi wholesale markets. All Bac Giang markets were strictly or predominantly populated by Cluster V.1 or V.2 sellers, or both (Fig. 2). Cluster V.3 was the only, or the predominant, seller profile in 13 of the 17 Ha Noi retail and mixed markets. This seller profile was, however, absent in 2 markets located in peri-urban areas, far from the main urban centres. Contrary to other market groups, Ha Noi wholesale markets were highly heterogeneous in terms of seller composition. However, when considering the proportion of poultry traded by each seller profile in each market (Fig. S1), large-scale sellers (Cluster V.4) were predominant in all Ha Noi wholesale markets but 1. The market location was, therefore, a good predictor of the seller composition.

Cambodian sellers were classified into 2 clusters. Cluster C.1 sellers spent little time in LBMs and either rarely, or never, had a surplus. In contrast, most Cluster C.2 sellers spent all day in LBMs and reported high surpluses. The median length of time spent in LBMs was 4 hours for Cluster C.1, and 11 hours for Cluster C.2 sellers. Most Cluster C.1 sellers (58%, n = 26) never had any unsold poultry at the end of the market day, whereas all Cluster C.2 sellers but 1 reported surpluses. Whilst most Cluster C.1 sellers were supplied by farmers, most Cluster C.2 sellers were supplied by traders. Contrary to Viet Nam, all supplying farms were backyard farms (<50 birds), and none of the Cambodian sellers visited other markets to buy or sell poultry.

Cambodian seller profiles were also associated with market groups, with 85% (n = 26) Cluster C.1 sellers operating in peri-urban markets and 86% (n = 28) Cluster C.2 sellers operating in urban markets. Three of 4 peri-urban markets were exclusively populated by Cluster C.1 sellers, and Cluster C.2 predominated in 3 of 4 urban markets.

Chicken sales peaked in Viet Nam and Cambodia during the Tet and the Chinese New Year (late January or early February), respectively, with 72% (n = 340) and 96% (n = 54) sellers reporting an increase in chicken sales by 100% on average. Likewise, the number of sellers operating at markets increased. Sellers did not report any other changes in their practices during these periods.

## Discussion

Despite considerable variation in poultry management practices between sellers, some patterns were evident such that seller profiles of epidemiological importance could be identified. The high surplus frequency and volume reported by Clusters V.3 sellers (medium-scale sellers with high surplus) increased the time spent by birds in the LBM system. Moreover, their low supply frequency and the practice of purchasing birds the day before offering them for sale extended the time spent by birds in the sellers' flocks. This created opportunities for newly purchased birds to mix with unsold birds brought back from LBMs. These practices would make infection of susceptible birds more likely. This seller profile would thus be at high risk for contributing to the maintenance of HPAIV H5N1 in LBMs.

In contrast, surplus and supply features exhibited by Cluster V.2 (medium-scale sellers with low surplus) and V.4 sellers (large-scale sellers) meant that their birds spent little time in LBMs, limiting their potential to sustain virus circulation. However, these seller profiles may still play an active role in virus spread. A substantial proportion of Cluster V.2 sellers were mobile, visiting several markets to sell and/or purchase poultry. This could enable them to spread infection between LBMs. If infectious birds are introduced into LBMs populated by Cluster V.4 sellers, characterized by large sale volumes and long periods of time spent at market per day, infection of susceptible birds could result. Although these newly infected birds are unlikely to remain in LBMs for a sufficient period of time to infect others, their sale to traders operating in other LBMs could lead to virus spread between LBMs. Indeed, most large-scale sellers (V.4) operated in Ha Noi wholesale markets, from which a substantial part of the poultry population was then sold to retail market sellers (data not shown). In contrast, Cluster V.1 sellers (farmers and irregular sellers) were unlikely to play a major role in spatial virus spread as they only traded in markets located in their vicinity.

The distribution of seller profiles was associated with market groups. Therefore, knowing the type and location of a given LBM provides a good indicator of its seller profile composition, the proportion of poultry sold by each seller profile and, thus, the market's risk of sustaining HPAIV H5N1 circulation. LBMs located in the rural province of Bac Giang probably play a limited role in virus perpetuation. By contrast, most Ha Noi retail and mixed markets were dominated by Cluster V.3 sellers, which could permit virus maintenance. However, the low number of sellers and the low volume of sales might increase the risk of stochastic extinction of viruses, even with frequent surpluses. Two LBMs of this group were predominantly or exclusively populated by Cluster V.2 sellers and were located in districts which shared key features with Bac Giang province: rural areas with a large number of poultry farms. In contrast, other Ha Noi retail and mixed markets were located in, or close to, urban centres. This urban tropism of sellers at higher risk of maintaining virus circulation was also observed in Cambodia. The markets located in Phnom Penh or its outskirts were almost exclusively populated by sellers with high surpluses (C.2), similar to Vietnamese Cluster V.3 sellers, whilst Cambodian Cluster C.1 sellers, unlikely to allow virus maintenance, operated in provinces other than Phnom Penh.

Basic information on market type and location could be easily collected from each LBM to aid the identification of markets at high risk of virus maintenance, where risk mitigation strategies should be implemented. This information could be directly collected from market managers and would not require a labor-intensive survey. The implementation of strategies aiming at breaking virus amplification cycles in all markets is unnecessary, and also impractical given that LBMs are ubiquitous. Targeting control measures to a few selected markets would not only reduce the overall cost, but would also allow closer monitoring and proper implementation. Simple hygiene measures and culling of unsold birds may be very effective in breaking the virus amplification cycle [11], [34], [35]. Successfully implemented in Hong Kong, such measures would, however, need to be adapted to each local setting in order to minimize their negative impact on trade.

HPAIV H5N1 has been isolated in northern Vietnamese LBMs [36], [37], and poultry trade is suspected to spread the infection in Cambodia [38]. HPAIV H5N1 is likely to circulate in the study population. However, only the potential of LBMs to become virus reservoirs has been assessed in this survey. Complementing the findings with virological sampling of markets would be necessary to conclude that these high risk LBMs are truly virus reservoirs. Moreover, since the sampling frame is not representative of the population as a whole but of the largest markets in specific regions, inferences about the study population are necessarily limited. When asked to name the most populated markets, traders were more likely to name markets where they regularly operated, or of which they had personal knowledge, for example by being in their vicinity. However, as traders interviewed in Bac Giang province often named the same markets, regardless of the district where the interview took place, it is likely that most of the largest markets were identified. Three markets located in Bac Giang city were among the most commonly named markets, but authorization to visit was not possible as poultry sales at these sites had been officially prohibited. In Ha Noi province, some large retail and mixed markets may have been missed, as only a few districts were visited. However, the retail and mixed markets identified in Ha Noi were likely to be similar to those not identified, given similar population densities and housing.

In conclusion, this study was able to identify specific profiles of live bird sellers in Viet Nam and Cambodia which could play a key role in virus perpetuation. Moreover, the type and the location of an LBM could be a good predictor of its seller profile composition and, thus, of its potential for sustaining virus circulation. Therefore, results suggest that control strategies aiming at preventing HPAIV H5N1 maintenance in LBMs could potentially be targeted towards specific high risk LBM groups. This is of particular importance in resource-scarce countries with extensively developed LBM systems.

## Supporting Information

Figure S1 **Distribution of the number of poultry traded by seller clusters across markets and market groups.** For each market group, a barplot (on the left) shows the proportion of the poultry flow (number of poultry sold) traded by each seller cluster in the market group, and a plot (on the right) shows the distribution of its markets according to the proportion of the poultry flow traded by each seller cluster in each market. Where the number of markets in a group is greater than 5, box plots are shown; otherwise each market (circle) and the median (line) are presented.
(TIF)

Text S1 **Supplementary information includes further details on the approach used to construct a typology of traders, and on the results of the multivariate analysis and hierarchical cluster analysis.**
(DOC)

## References

[1] Xu X, Subbarao, Cox NJ, Guo Y (1999). Genetic characterization of the pathogenic influenza A/Goose/Guangdong/1/96 (H5N1) virus: similarity of its hemagglutinin gene to those of H5N1 viruses from the 1997 outbreaks in Hong Kong.. Virology.

[2] Li C, Hatta M, Nidom CA, Muramoto Y, Watanabe S (2010). Reassortment between avian H5N1 and human H3N2 influenza viruses creates hybrid viruses with substantial virulence.. Proc Natl Acad Sci U S A.

[3] Shortridge KF (1999). Poultry and the influenza H5N1 outbreak in Hong Kong, 1997: abridged chronology and virus isolation.. Vaccine.

[4] Abdelwhab EM, Selim AA, Arafa A, Galal S, Kilany WH (2010). Circulation of avian influenza H5N1 in live bird markets in Egypt.. Avian Dis.

[5] Indriani R, Samaan G, Gultom A, Loth L, Indryani S (2010). Environmental sampling for avian influenza virus A (H5N1) in live-bird markets, Indonesia.. Emerg Infect Dis.

[6] Webster RG (2004). Wet markets–a continuing source of severe acute respiratory syndrome and influenza?. Lancet.

[7] Soares Magalhaes RJ, Ortiz-Pelaez A, Thi KL, Dinh QH, Otte J (2010). Associations between attributes of live poultry trade and HPAI H5N1 outbreaks: a descriptive and network analysis study in northern Vietnam.. BMC Vet Res.

[8] Kim HR, Park CK, Lee YJ, Woo GH, Lee KK (2010). An outbreak of highly pathogenic H5N1 avian influenza in Korea, 2008.. Vet Microbiol.

[9] Van Kerkhove MD, Mumford E, Mounts AW, Bresee J, Ly S (2011). Highly pathogenic avian influenza (H5N1): pathways of exposure at the animal-human interface, a systematic review.. PLoS One.

[10] Wan XF, Dong L, Lan Y, Long LP, Xu C (2011). Indications that live poultry markets are a major source of human H5N1 influenza virus infection in China.. J Virol.

[11] Fournié G, Guitian FJ, Mangtani P, Ghani AC (2011). Impact of the implementation of rest days in live bird markets on the dynamics of H5N1 highly pathogenic avian influenza.. J R Soc Interface.

[12] Amonsin A, Choatrakol C, Lapkuntod J, Tantilertcharoen R, Thanawongnuwech R (2008). Influenza virus (H5N1) in live bird markets and food markets, Thailand.. Emerg Infect Dis.

[13] Choi YK, Seo SH, Kim JA, Webby RJ, Webster RG (2005). Avian influenza viruses in Korean live poultry markets and their pathogenic potential.. Virology.

[14] Chen H, Smith GJ, Li KS, Wang J, Fan XH (2006). Establishment of multiple sublineages of H5N1 influenza virus in Asia: implications for pandemic control.. Proc Natl Acad Sci U S A.

[15] Chen J, Fang F, Yang Z, Liu X, Zhang H (2009). Characterization of highly pathogenic H5N1 avian influenza viruses isolated from poultry markets in central China.. Virus Res.

[16] Guan Y, Peiris JS, Lipatov AS, Ellis TM, Dyrting KC (2002). Emergence of multiple genotypes of H5N1 avian influenza viruses in Hong Kong SAR.. Proc Natl Acad Sci U S A.

[17] Liu M, He S, Walker D, Zhou N, Perez DR (2003). The influenza virus gene pool in a poultry market in South central china.. Virology.

[18] Ge FF, Zhou JP, Liu J, Wang J, Zhang WY (2009). Genetic evolution of H9 subtype influenza viruses from live poultry markets in Shanghai, China.. J Clin Microbiol.

[19] Lee HJ, Kwon JS, Lee DH, Lee YN, Youn HN (2010). Continuing evolution and interspecies transmission of influenza viruses in live bird markets in Korea.. Avian Dis.

[20] OIE (2011). World Organisation for Animal Health. H5N1 Notified in Domestic Poultry 2003–2010.. Paris: OIE (World Organization for Animal Health).

[21] ProMED-mail (2008). Avian Influenza (122): Cambodia, China, India, Taiwan (suspected).. http://www.promedmail.org.

[22] Van Kerkhove MD, Vong S, Guitian J, Holl D, Mangtani P (2009). Poultry movement networks in Cambodia: implications for surveillance and control of highly pathogenic avian influenza (HPAI/H5N1).. Vaccine.

[23] Meleigy M (2007). Egypt battles with avian influenza.. Lancet.

[24] Peyre M, Samaha H, Makonnen YJ, Saad A, Abd-Elnabi A (2009). Avian influenza vaccination in Egypt: Limitations of the current strategy.. J Mol Genet Med.

[25] Minh PQ, Morris RS, Schauer B, Stevenson M, Benschop J (2009). Spatio-temporal epidemiology of highly pathogenic avian influenza outbreaks in the two deltas of Vietnam during 2003–2007.. Prev Vet Med.

[26] General Statistics Office of Vietnam (2010). Population and population density in 2009 by province.. http://www.gso.gov.vn/default_en.aspx?tabid=467&idmid=3&ItemID=9882.

[27] General Statistics Office of Vietnam (2010). Number of poultry by province 2009.. http://www.gso.gov.vn/Default.aspx?tabid=217.

[28] Hulse-Post DJ, Sturm-Ramirez KM, Humberd J, Seiler P, Govorkova EA (2005). Role of domestic ducks in the propagation and biological evolution of highly pathogenic H5N1 influenza viruses in Asia.. Proc Natl Acad Sci U S A.

[29] Pages J (2004). Analyse factorielle de donnees mixtes.. Revue de statistique applique.

[30] Bertrand F, Maumy M, Fussler L, Kobes N, Savary S (2007). Using Factor Analyses to explore data generated by the National Grapevine Wood Diseases Survey.. Case Studies in Business, Industrial or Governmental Statistics.

[31] Manly BFJ (2005). Multivariate Statistical Methods: A Primer: Chapman & Hall/CRC Press..

[32] R Development Core Team (2010). A Language and Environment for Statistical Computing and Graphics.. Vienna.

[33] Lê S, Josse J, Husson F (2008). FactoMineR: An R Package for Multivariate Analysis.. Journal of Statistical Software.

[34] Kung NY, Guan Y, Perkins NR, Bissett L, Ellis T (2003). The impact of a monthly rest day on avian influenza virus isolation rates in retail live poultry markets in Hong Kong.. Avian Dis.

[35] Lau EHY, Leung YHC, Zhang LJ, Cowling BJ, Mak SP (2007). Effect of Interventions on Influenza A (H9N2) Isolation in Hong Kong's Live Poultry Markets, 1999–2005.. Emerging Infectious diseases.

[36] Nguyen DC, Uyeki TM, Jadhao S, Maines T, Shaw M (2005). Isolation and characterization of avian influenza viruses, including highly pathogenic H5N1, from poultry in live bird markets in Hanoi, Vietnam, in 2001.. Journal of Virology.

[37] Davis CT, Balish AL, O'Neill E, Nguyen CV, Cox NJ (2010). Detection and characterization of clade 7 high pathogenicity avian influenza H5N1 viruses in chickens seized at ports of entry and live poultry markets in Vietnam.. Avian Dis.

[38] Buchy P, Fourment M, Mardy S, Sorn S, Holl D (2009). Molecular epidemiology of clade 1 influenza A viruses (H5N1), southern Indochina peninsula, 2004–2007.. Emerg Infect Dis.

