# Supplementary figures and images for: Identifying Live Bird Markets with the Potential to Act as Reservoirs of Avian Influenza A (H5N1) Virus: A Survey in Northern Viet Nam and Cambodia
Source: PLoS One. 2012 Jun 4;7(6):e37986. doi: 10.1371/journal.pone.0037986 (PMC3366999; doi:10.1371/journal.pone.0037986)

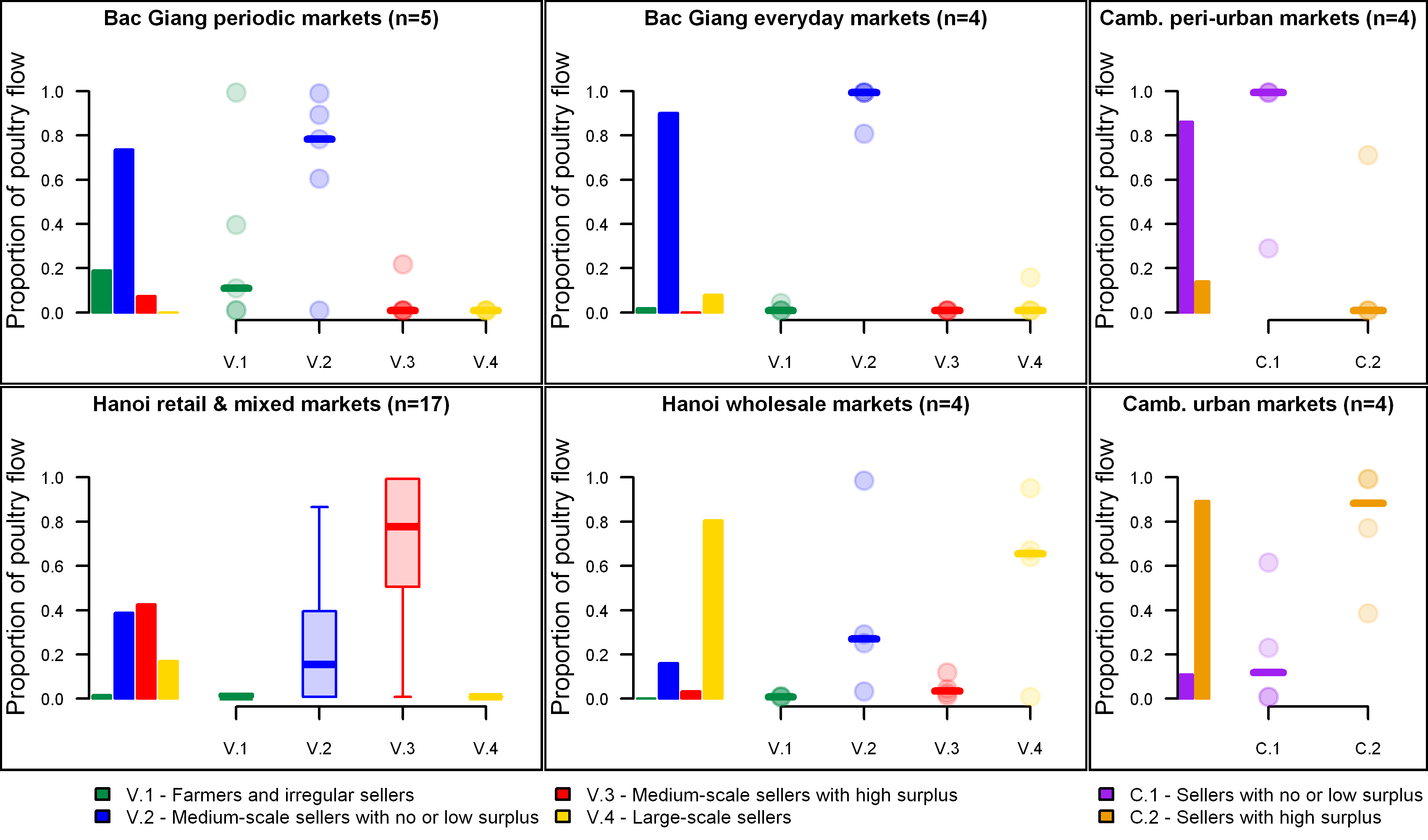

Supplement: Figure S1 — Distribution of the number of poultry traded by seller clusters across markets and market groups. For each market group, a barplot (on the left) shows the proportion of the poultry flow (number of poultry sold) traded by each seller cluster in the market group, and a plot (on the right) shows the distribution of its markets according to the proportion of the poultry flow traded by each seller cluster in each market. Where the number of markets in a group is greater than 5, box plots are shown; otherwise each market (circle) and the median (line) are presented. (TIF) [file pone.0037986.s001.tif]
