# Supplementary material for: Identifying Live Bird Markets with the Potential to Act as Reservoirs of Avian Influenza A (H5N1) Virus: A Survey in Northern Viet Nam and Cambodia
Source: PLoS One. 2012 Jun 4;7(6):e37986. doi: 10.1371/journal.pone.0037986 (PMC3366999; doi:10.1371/journal.pone.0037986)
Supplement: Text S1 — Supplementary information includes further details on the approach used to construct a typology of traders, and on the results of the multivariate analysis and hierarchical cluster analysis. (DOC) [file pone.0037986.s002.doc]

**Text S1**

Supplementary information includes further details on the approach used to construct a typology of traders, and on the results of the multivariate analysis and hierarchical cluster analysis.

**Factor analysis for mixed data**

Multivariate statistical methods can reduce the dimensions of multivariate data and create a smaller number of synthetic factors accounting for most data variability. A number of correlated variables are transformed into a smaller number of uncorrelated factors. Principal component analysis (PCA) can be used for numerical variables and multiple correspondence analysis (MCA) for categorical variables [1]. When both numerical and categorical variables are present, factor analysis for mixed data (FAMD) allows construction of a transformation with both numerical and categorical variables without necessitating the recoding of numerical variables as categorical variables. Numerical variables are transformed as in a normalized PCA (i.e. numerical variables are scaled prior to their transformation to have a mean of 0 and a variance of 1) and categorical variables as in an MCA. This is equivalent to a multiple factor analysis (MFA) in which each variable set is composed by only 1 variable [2].

Within the variable space, the first factor is the axis accounting for as much data variability as possible. Each succeeding factor captures the maximum possible amount of data variability, not explained by former factors. Consider the *s^th^* factor *z_s_* associated with the eigenvalue *λ_s_*. There are *K* numerical variables *v_k_*, with $1\leq k\leq K$, and *Q* categorical variables *V_q_*, with $1\leq q\leq Q$. Then the eigenvalue *λ_s_* associated with the factor *z_s_* is given by the equation:

$\lambda_{s}=\sum_{k} r^{2}\left(z_{s},v_{k}\right)+\sum_{q} \eta^{2}\left(z_{s},V_{q}\right)$ (1)

The first term is the sum of the squared correlation coefficients between factor *z_s_* and all numerical variables *v_k_*, and the second term is the sum of the squared correlation ratio between factor *z_s_* and all categorical variables *V_q_*. The eigenvalue is thus a measure of the amount of variance in the data that is explained by the related factor. The factor *z_s_* is obtained by maximizing this expression, to the condition that it is not correlated for the data to factors previously defined.

A categorical variable *V_q_* is represented by the set of its indicator functions. An indicator function is defined for each categorical value. It is binary, equal to 1 for observations with this categorical value and 0 for others. Each indicator function is weighted by the proportion of observations that take that categorical value. Therefore, during the process of construction of the axis, the influences of all the variables are balanced, and do not depend on the number of levels of the categorical ones [3].

Each successive component contains a decreasing proportion of the total data variability. It is expected that variances of most factors are negligible, and the data variability can be described by a small number of factors. Therefore, only factors catching a substantial part of data variance are kept. Eigenvalues should be separated by a break on the scree plot. A sharp drop represents the amount of information captured by selected factors and separates small and large eigenvalues. Moreover, factors catching less variability than one of the initial variables were disregarded.

#### Procedure

Some Poultry management variables (main text, Table 1) were specific to chicken or duck sales, yet all sellers did not sell both poultry types. Three seller categories were thus defined and analyzed separately: chicken sellers (i.e. sellers trading only chickens), duck sellers (i.e. sellers trading only ducks) and sellers of both species (i.e. sellers trading both chickens and ducks). Vietnamese and Cambodian sellers were also differentiated.

**Results**

**Multivariate analysis**

The FAMD was performed for Vietnamese and Cambodian sellers of both species (chickens and ducks), Vietnamese and Cambodian chicken sellers, and Vietnamese duck sellers. For all analyses, the 2 first factors were selected, except for Cambodian sellers of both species for which 3 factors were kept (Table S1.1).

Table S1.1. Percentage of variance explained by factors chosen for each FAMD performed for Vietnamese and Cambodian sellers.

| Seller category | 1^st^ Factor | 2^nd^ Factor | 3^rd^ Factor |
| --- | --- | --- | --- |
| Vietnamese both species sellers | 18% | 15% | - |
| Vietnamese chicken sellers | 31% | 25% | - |
| Vietnamese duck sellers | 28% | 19% | - |
| Cambodian both species sellers | 28% | 19% | 14% |
| Cambodian chicken sellers | 40% | 24% | - |

The distribution of sellers as a function of factor values is presented in Fig. S1.1 and S1.2. Sellers with a high score for each of these factors are described below. The converse is true for sellers with a low score.

*Viet Nam, sellers of both species*

Factor 1: Sellers spent long periods of time at market and had a surplus every day, with a high proportion of unsold chickens and ducks. They were not farmers, did not buy birds everyday and kept birds at home before going to market.

Factor 2: Sellers traded chickens occasionally and ducks everyday. The volume of sales was high for both poultry types.

*Viet Nam, chicken sellers*

Factor 1: Sellers operated at markets every day and throughout the day, with a high volume of sales. They were supplied with birds every day, which were taken directly to the market after purchase.

Factor 2: Sellers had a surplus everyday, with a high proportion of unsold chickens. Birds were not bought every day and were kept at home before being brought to market.

*Viet Nam, duck sellers*

Factor 1: Sellers traded every day at the market, throughout the day. They were not farmers, sold a high number of ducks, and never had any surplus.

Factor 2: Sellers had a surplus every day, with a high proportion of unsold ducks. They were not farmers, they did not buy birds every day and birds were kept at home before being brought to market.

*Cambodia, sellers of both species*

Factor 1: Sellers spent all day at market, had a surplus every day, with a high proportion of unsold chickens and ducks.

Factor 2: In the event of a surplus, the proportion of unsold chickens was low. Sellers bought birds every day.

Factor 3: Sellers traded a high number of chickens, they did not buy birds everyday and birds were kept at home before being brought to market.

*Cambodia, chicken sellers*

Factor 1: Sellers had a surplus every day, with a high proportion of unsold chickens.

Factor 2: Sellers traded a high number of chickens. They were supplied with birds everyday.

#### Hierarchical cluster analysis

Within each of the 3 categories (i.e. chicken, duck, both species), Vietnamese sellers were partitioned into 4 clusters. These partitions were similar between categories (i.e. each of the 4 clusters showed similar features across the 3 categories). Therefore, Vietnamese sellers were considered to be divided into 4 clusters.

Cambodian sellers of both species were divided into 2 main clusters, of which 1 could then be subdivided into 2 sub-clusters. Chicken sellers were divided into 2 clusters in a similar way to those of the sellers of both species. Due to these similarities in clustering features between sellers of both species and chicken sellers, Cambodian sellers could, thus, be considered to be partitioned into 2 main clusters. One of these clusters was subdivided into 2 sub-clusters.

The impact of the choice of the distance (i.e. Manhattan or Euclidean) on the results was explored. Using Euclidean distances, the same partitions were achieved for all analyses, except for Vietnamese sellers of both species. These sellers were clustered into 3 groups, of which 1 was the merging of 2 of the 4 clusters obtained using Manhattan distances, with only 4 sellers being misclassified between these 2 partitions. The distribution of poultry management variables for each seller category (i.e. both species, chicken, duck) and seller type are presented in Table S1.2 to S1.4.


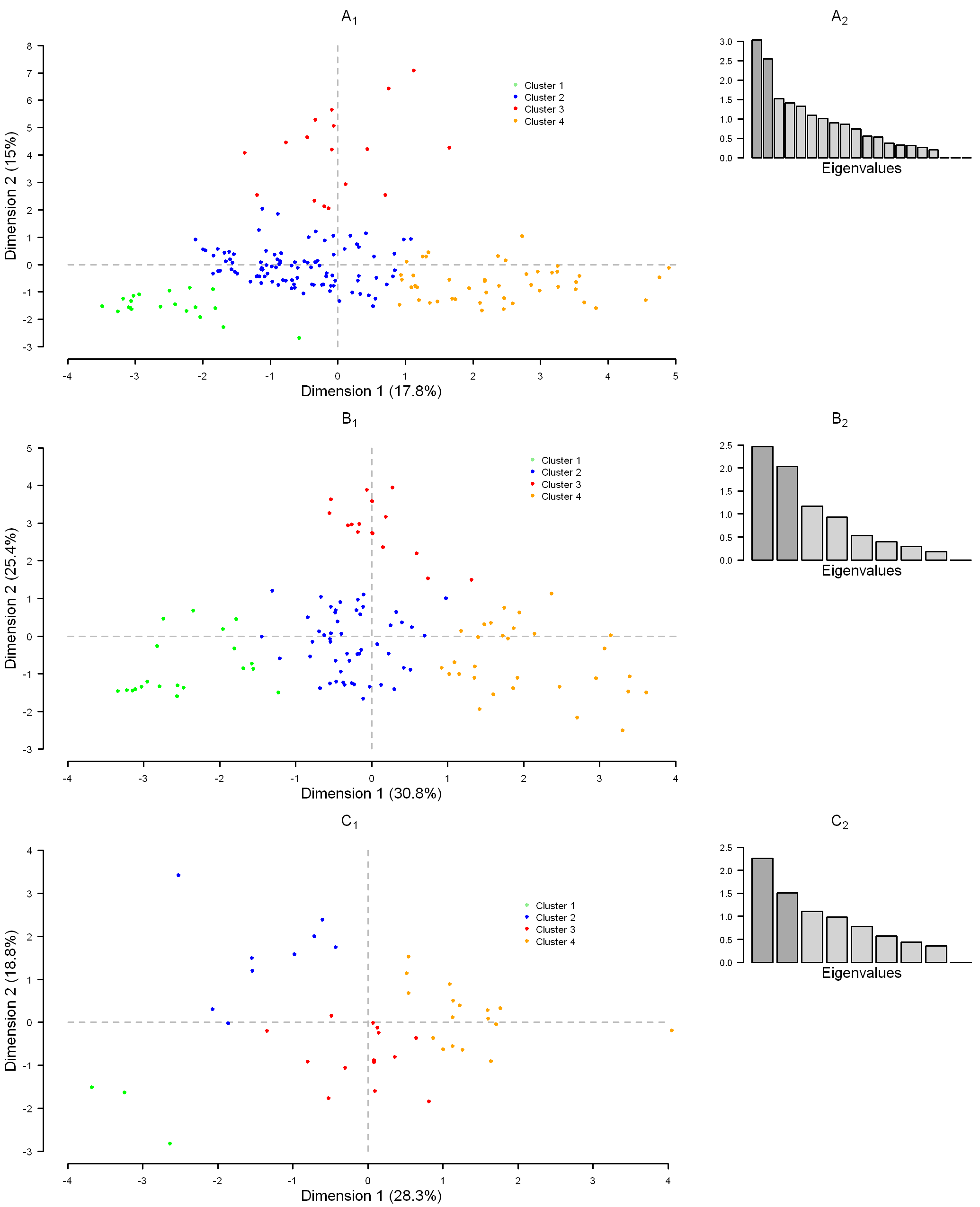


Figure S1.1. Distribution of Vietnamese sellers as a function of factors identified in each FAMD and diagrams of eigenvalues.

(1): Distribution of sellers as a function of the 2 main factors. (2): diagrams of the eigenvalues, the darkest eigenvalues are those of chosen factors. (A): Both Species Sellers; (B): Chicken sellers; (C): Duck sellers. Green: Cluster V.1; Blue: Cluster V.2; Red: Cluster V.3; Orange: Cluster V.4.


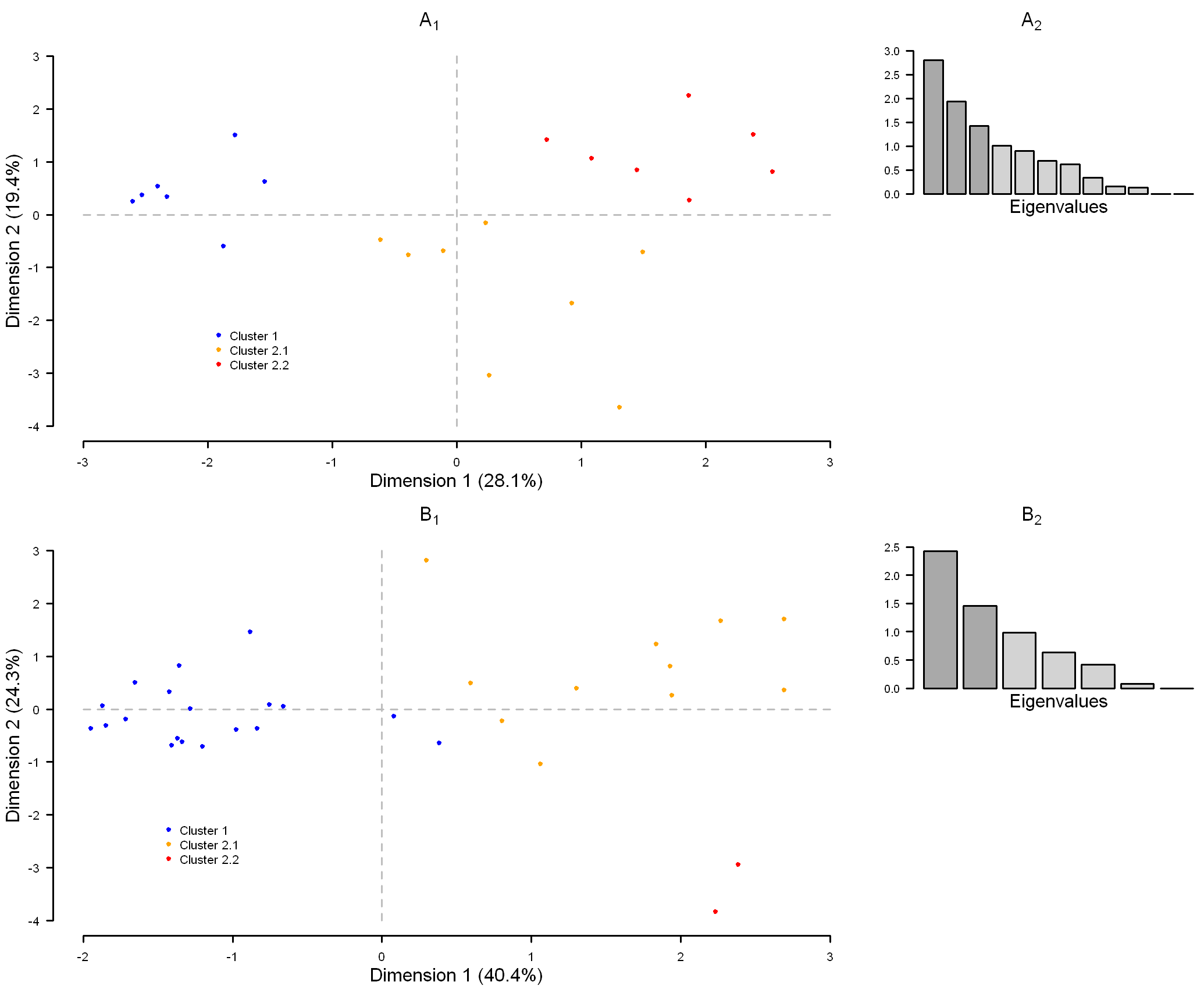


Figure S1.2. Distribution of Cambodian sellers as a function of factors identified in each FAMD and diagrams of eigenvalues.

(1): Distribution of sellers as a function of the 2 main factors. (2): diagrams of the eigenvalues, the darkest eigenvalues are those of chosen factors. (A): Both Species Sellers; (B): Chicken sellers. Blue: Cluster C.1; Red: Cluster C.2.1; Orange: Cluster C.2.2.

Table S1.2. Features of Vietnamese sellers in Cluster V.1 and V.2.

Data are median (inter-quartile range), or n (%). Both sp: sellers of both species; CK: chicken sellers; DK: duck sellers; Ev: Every time; S: Store purchased birds at home before offering them for sale; nS: Do not store purchased birds at home.

|  | **V.1 – Farmers and occasional sellers** | | | **V.2 – medium sellers with no or low surplus** | | |
| --- | --- | --- | --- | --- | --- | --- |
|  | **Both sp** | **CK** | **DK** | **Both sp** | **CK** | **DK** |
| ***N*** | 20 | 20 | 3 | 97 | 51 | 14 |
| Days/Month | 12 (10-12) | 8 (4-13) | 4 (3-6) | 30 (24-30) | 30 (25-30) | 19 (10-24) |
| Time/Day (hours) | 2 (1-3) | 3 (2-4) | 3 (3-3) | 3 (3-4) | 5 (3-5) | 6 (4-7) |
| CK – Never | 0 (0%) | 0 (0%) | - | 0 (0%) | 0 (0%) | - |
| Seasonal | 0 (0%) | 0 (0%) | - | 0 (0%) | 0 (0%) | - |
| Sometimes | 0 (0%) | 0 (0%) | - | 2 (2%) | 0 (0%) | - |
| Often | 3 (15%) | 0 (0%) | - | 20 (21%) | 0 (0%) | - |
| Always | 17 (85%) | 20 (100%) | - | 74 (76%) | 51 (100%) | - |
| No. CK | 7 (6-9) | 9 (4-10) | - | 19 (10-30) | 20 (13.5-67.5) | - |
| DK – Never | 0 (0%) | - | 0 (0%) | 0 (0%) | - | 0 (0%) |
| Summer | 7 (35%) | - | 0 (0%) | 40 (41%) | - | 0 (0%) |
| Sometimes | 12 (60%) | - | 0 (0%) | 31 (32%) | - | 0 (0%) |
| Often | 1 (5%) | - | 0 (0%) | 19 (20%) | - | 0 (0%) |
| Always | 0 (0%) | - | 3 (100%) | 7 (7%) | - | 14 (100%) |
| No. DK | 6 (4-8) | - | 8 (8-9) | 14 (7-24) | - | 50 (43-69) |
| Supply – Never | 20 (100%) | 17 (85%) | 3 (100%) | 0 (0%) | 4 (8%) | 0 (0%) |
| Not Ev – nS | 0 (0%) | 0 (0%) | 0 (0%) | 0 (0%) | 0 (0%) | 0 (0%) |
| Not Ev – S | 0 (0%) | 0 (0%) | 0 (0%) | 2 (2%) | 9 (18%) | 0 (0%) |
| Ev – nS | 0 (0%) | 2 (10%) | 0 (0%) | 70 (72%) | 23 (45%) | 5 (36%) |
| Ev – S | 0 (0%) | 1 (5%) | 0 (0%) | 25 (26%) | 15 (29%) | 9 (64%) |
| Reported surplus | 8 | 8 | 2 | 85 | 35 | 8 |
| Surplus frequency | 0% (0%-17%) | 0% (0%-25%) | 50% (25%-50%) | 25% (7%-43%) | 14% (0%-40%) | 7% (0%-25%) |
| Proportion of unsold CK | 13% (9%-17%) | 16% (8%-24%) | - | 11% (7%-17%) | 10% (7%-19%) | - |
| Proportion of unsold DK | 13% (13%-14%) | - | 5% (4%-5%) | 8% (5%-12%) | - | 5% (4%-6%) |

Table S1.3. Features of Vietnamese sellers in Cluster V.3 and V.4.

Data are median (inter-quartile range), or n (%). Both sp: sellers of both species; CK: chicken sellers; DK: duck sellers; Ev: Every time; S: Store purchased birds at home before offering them for sale; nS: Do not store purchased birds at home.

|  | **V.3 – medium sellers with high surplus** | | | **V.4 – large sellers** | | |
| --- | --- | --- | --- | --- | --- | --- |
|  | **Both sp** | **CK** | **DK** | **Both sp** | **CK** | **DK** |
| ***N*** | 46 | 16 | 9 | 17 | 30 | 17 |
| Days/Month | 30 (25-30) | 28 (24-30) | 15 (10-15) | 30 (20-30) | 30 (30-30) | 28 (25-30) |
| Time/Day (hours) | 5 (4-11) | 4 (4-5) | 5 (3-6) | 8 (7-13) | 12 (10-13) | 10 (8-12) |
| CK – Never | 0 (0%) | 0 (0%) | - | 0 (0%) | 0 (0%) | - |
| Seasonal | 0 (0%) | 0 (0%) | - | 6 (35%) | 0 (0%) | - |
| Sometimes | 0 (0%) | 0 (0%) | - | 6 (35%) | 0 (0%) | - |
| Often | 28 (61%) | 0 (0%) | - | 2 (12%) | 0 (0%) | - |
| Always | 18 (39%) | 16 (100%) | - | 3 (18%) | 30 (100%) | - |
| No. CK | 10 (5-20) | 7 (5-10) |  | 75 (50-300) | 289 (150-488) | - |
| DK – Never | 0 (0%) | - | 0 (0%) | 0 (0%) | - | 0 (0%) |
| Summer | 4 (9%) | - | 0 (0%) | 0 (0%) | - | 0 (0%) |
| Sometimes | 29 (63%) | - | 0 (0%) | 4 (24%) | - | 0 (0%) |
| Often | 11 (24%) | - | 0 (0%) | 4 (24%) | - | 0 (0%) |
| Always | 2 (4%) | - | 9 (100%) | 9 (53%) | - | 17 (100%) |
| No. DK | 7 (5-12) | - | 33 (12-100) | 100 (67-167) | - | 167 (100-200) |
| Supply – Never | 0 (0%) | 0 (0%) | 0 (0%) | 0 (0%) | 0 (0%) | 0 (0%) |
| Not Ev – nS | 3 (7%) | 0 (0%) | 0 (0%) | 0 (0%) | 0 (0%) | 0 (0%) |
| Not Ev – S | 16 (35%) | 12 (75%) | 1 (11%) | 0 (0%) | 0 (0%) | 1 (6%) |
| Ev – nS | 15 (33%) | 0 (0%) | 6 (67%) | 16 (94%) | 29 (97%) | 14 (82%) |
| Ev – S | 12 (26%) | 4 (25%) | 2 (22%) | 1 (6%) | 1 (3%) | 2 (12%) |
| Reported surplus | 46 (100%) | 16 (100%) | 9 (100%) | 15 (88%) | 27 (90%) | 12 (71%) |
| Surplus frequency | 86%  (69%-100%) | 100%  (100%-100%) | 67%  (50%-75%) | 20%  (10%-43%) | 43%  (29%-86%) | 7%  (0%-29%) |
| Proportion of unsold CK | 29% (22%-41%) | 37% (28%-48%) | - | 8% (6%-11%) | 9% (6%-15%) | - |
| Proportion of unsold DK | 17% (13%-25%) | - | 17% (9%-25%) | 6% (4%-8%) | - | 8% (6%-10%) |

Table S1.4. Features of Cambodian sellers in Clusters C.1 and C.2.

Data are median (inter-quartile range), or n (%). Both sp: sellers of both species; CK: chicken sellers; DK: duck sellers; Ev: Every time; S: Store purchased birds at home before offering them for sale; nS: Do not store purchased birds at home.

|  | **Cluster C.1: no or low surplus** | | **Cluster C.2: High surplus** | | | |
| --- | --- | --- | --- | --- | --- | --- |
|  | **Both sp** | **CK** | **C.2.1 Both sp** | **C.2.1 CK** | **C.2.2 Both sp** | **C.2.2 CK** |
| ***N*** | 7 | 19 | 8 | 2 | 7 | 11 |
| Days/Month | 30 (30-30) | 30 (30-30) | 30 (30-30) | 30-30 | 30 (30-30) | 30 (30-30) |
| Time/Day (hours) | 3(2-4) | 4 (3-6) | 11 (5-12) | 4-11 | 11 (11-12) | 11 (10-12) |
| CK – Never | 0 (0%) | 0 (0%) | 0 (0%) | 0 (0%) | 0 (0%) | 0 (0%) |
| Seasonal | 0 (0%) | 0 (0%) | 0 (0%) | 0 (0%) | 0 (0%) | 0 (0%) |
| Sometimes | 0 (0%) | 0 (0%) | 0 (0%) | 0 (0%) | 0 (0%) | 0 (0%) |
| Often | 0 (0%) | 0 (0%) | 1 (13%) | 0 (0%) | 1 (14%) | 0 (0%) |
| Always | 7 (100%) | 19 (100%) | 7 (88%) | 2 (100%) | 6 (86%) | 11 (100%) |
| No. CK | 30 (30-50) | 10 (10-30) | 20 (16-26) | 13-13 | 50 (41-92) | 48 (25-77) |
| DK – Never | 0 (0%) | - | 0 (0%) | - | 0 (0%) | - |
| Summer | 0 (0%) | - | 0 (0%) | - | 0 (0%) | - |
| Sometimes | 7 (100%) | - | 8 (100%) | - | 4 (57%) | - |
| Often | 0 (0%) | - | 0 (0%) | - | 2 (29%) | - |
| Always | 0 (0%) | - | 0 (0%) | - | 1 (14%) | - |
| No. DK | 8 (6-10) | - | 4 (2-5) | - | 23 (15-30) | - |
| Supply – Never | 0 (0%) | 0 (0%) | 0 (0%) | 0 (0%) | 0 (0%) | 0 (0%) |
| Not Ev – nS | 0 (0%) | 0 (0%) | 2 (25%) | 2 (100%) | 0 (0%) | 0 (0%) |
| Not Ev – S | 0 (0%) | 0 (0%) | 1 (13%) | 0 (0%) | 0 (0%) | 0 (0%) |
| Ev – nS | 7 (100%) | 13 (68%) | 5 (63%) | 0 (0%) | 7 (100%) | 10 (91%) |
| Ev – S | 0 (0%) | 6 (32%) | 0 (0%) | 0 (0%) | 0 (0%) | 1 (9%) |
| Reported surplus | 1 (14%) | 10 (53%) | 8 (100%) | 2 (100%) | 7 (100%) | 10 (91%) |
| Surplus frequency | 0%  (0%-0%) | 7%  (0%-21%) | 100%  (43%-100%) | 43%-100% | 100%  (86% -100%) | 100%  (86%-100%) |
| Proportion of unsold CK | 20% (20%-20%) | 11% (11%-15%) | 26% (22%-32%) | 40%-40% | 17% (9%-28%) | 24% (21%-29%) |
| Proportion of unsold DK | 0% (0%-0%) | - | 10% (8%-12%) | - | 20% (19%-25%) | - |

**References**

1. Manly BFJ. Multivariate Statistical Methods: A Primer. Third edition ed: Chapman & Hall/CRC Press; 2005.

2. Pages J. Analyse factorielle de donnees mixtes. Revue de statistique applique. 2004;52(4):93-111.

3. Bertrand F, Maumy M, Fussler L, Kobes N, Savary S, Grosman J. Using Factor Analyses to explore data generated by the National Grapevine Wood Diseases Survey. Case Studies in Business, Industrial or Governmental Statistics. 2007;1(2):183-202.
